# Metabolic Abnormality and Sleep Disturbance are Associated with Clinical Severity of Patients with Schizophrenia

**DOI:** 10.7603/s40681-014-0006-1

**Published:** 2014-08-06

**Authors:** Chung-Chieh Hung, Chin-Chih Liao, Po-Lun Wu, Shin-Da Lee, Hsien-Yuan Lane

**Affiliations:** 1 Graduate Institute of Clinical Medical Science, China Medical University, Taichung, Taiwan; 2 Department of Psychiatry, China Medical University Hospital, Taichung, Taiwan; 3 Graduate Institute of Rehabilitation Science, China Medical University, Taichung, Taiwan; 4 Department of Physical Therapy, China Medical University Hospital, Taichung, Taiwan; 5 Institute of Clinical Medical Science, College of Medicine, China Medical University, No.91 Hsueh-Shih Road, Taichung, Taiwan

**Keywords:** Schizophrenia, Metabolic syndrome, Sleep disturbance, Slow wave sleep, Body weight, Neck circumference

## Abstract

Schizophrenic patients suffer from more metabolic or sleep problems. Little is known about risk factors. We recruited 17 patients with chronic schizophrenia from the rehabilitation center in a medical center in Taiwan and measured their demographic data, cognitive performance, and physical fitness, metabolic profiles and sleep parameters. They were divided into two groups according to clinical severity, then compared in terms of metabolic and sleep parameters.

Those with more severe symptomatology had more metabolic abnormality and shorter slow wave sleep (SWS). Our findings suggest clinical symptoms as linked with heavier body weight, wider neck circumference, elevated blood pressure, and shorter SWS. Further studies are warranted to confirm the preliminary finding and to elucidate the underlying mechanism

## 1. Introduction

Schizophrenia, a psychiatric disorder causing deterioration of cognitive and daily function, is associated with obesity and metabolic syndrome, rendering patients vulnerable to morbidity and mortality[1].^1^ Biological factors, lifestyle, and antipsychotics all contribute to obesity of patients[2], [3], which influences their sleep quality[4]. Prevalence of poor sleepers among schizophrenics is around 45%, related to adverse events of medication and accompanying depression and psychological distress [5], [6]. Metabolic abnormality and sleep disturbance seem correlated. Consequently, these patients reportedly have poor life quality; correlation between clinical symptoms and sleep quality remains unclear. We hypothesize patients with severe clinical symptoms as more likely to have metabolic abnormality and sleep disturbance.

## 2. Methods

Study was approved by China Medical University Hospital Institutional Review Board (IRB). All participants gave written informed consent.

### 2.1. Participants

We recruited 17 schizophrenic patients from the Rehabilitation Center of the China Medical University Hospital Psychiatric Department. All met criteria of schizophrenia, paranoid type, according to DSM-IV-TR[7]. We rated the subjects by Positive and Negative Syndrome Scale (PANSS) [8], with respective items scored from 1(absent) to 7(extreme severity). We rated their depressive symptoms by Hamilton Depression Rating Scale[9], and quality oflife by Quality ofLife Scale (QLS) [10]. Extrapyramidal symptoms were rated by Abnormal Involuntary Movement (AIMS) [11], Simpson-Angus (SAS) [12], and Barnes Rating Scale (BARS) [13]. Daily antipsychotic doses were recorderd as chlorpromazine equivalents [14],^14^ and daily benzodiazepine doses as diazepam equivalents [15].

Inclusion criteria included (1) schizophrenic patients stable under current antipsychotics and benzodiazepine for at least three months; (2) engaged in regular rehabilitation program for at least three months; (3) aged between 20 and 50; (4) Han Taiwanese who speak Chinese fluently and understand this study well

Exclusion criteria included histories of (1) cerebrovascular, cardiovascular, and metabolic disorders (stroke, hypertension, diabetes mellitus); (2) neurologic disorders like epilepsy and traumatic brain injury; (3) physical disability (eg, fractures); (4) current DSM-IV-TR diagnosis of substance dependence (such as nicotine); (5) a DSM-IV-TR diagnosis of mental retardation, and (6) acute suicide or aggressive behaviors and (7) regular exercise.

### 2.2 Cognitive performance testing

Schizophrenic patients show impaired cognitive function [16], [17]. Our study included trail making, semantic association of verbal fluency, maze, verbal and non-verbal working memory, instant word list, delay word list, instant and, delayed visual reproduction, and digit symbol coding, as conducted by well-trained psychologists.

### 2.3 Cardiometabolic parameters and physical fitness

Patients’ weight and height, body mass index (BMI), neck circumference (NC), waist circumference, hip circumference, and waist-hip ratio (WHR) were recorded. Body fat was assessed by Omron body fat scale. Physical fitness was gauged according to a profile distributed by Bureau of Health Promotion, Department of Health, Taiwan. First, we checked sit-up frequency in one minute. Second, they underwent three-minute 35-centimeter-ladder climbing. We checked post-exercise heart rate (PEHR) at the end of the first minute (PEHR1), the second minute (PEHR2), and the third minute (PEHR3) while they stopped exercise. PEHR2 and PEHR3 were checked via similar method with total climbing time recorded.

Heart rate, systolic and diastolic blood pressure were measured before blood examination. Blood samples were taken at 8:00 a.m. after a 12-hour overnight fast, with subjects’ blood withdrawn from an antecubital vein to measure plasma levels of glucose, insulin, total cholesterol, triglyceride, HDL-cholesterol, LDL-cholesterol, and cortisol. Insulin was quantified by chemiluminescent immunoassay sandwich method, and serum glucose by glucose-oxidase-based assay. We rated insulin resistance by homeostasis model assessment of insulin resistance (HOMA-IR), assessed by the formula of fasting insulin (μU/ml) x fasting glucose (mg/dl)/405 [18].

The presence of metabolic syndrome was recorded as defined by National Cholesterol Education Program (NCEP) guidelines: waist circumference ≧102 cm (male) and ≧ 88 cm (female), triglyceride (TG) ≧ 150 mg/dl, HDL-cholesterol < 40 mg/dL (male) and < 50 mg/dL (female), blood pressure ≧130/85 mmHg, and fasting glucose ≧ 110 mg/dl[19]. Metabolic syndrome index was summated by the above criteria.

### 2.4 Sleep measurement

Sleep rating scales were self-recorded by all subjects preceding polysomnography examination: Pittsburgh Sleep Quality Index (PSQI) [20], Insomnia Severity Index (ISI) [21], Epworth Sleepness Scale (ESS) [22], and Pre-Sleep Arousal Scale (PSAS) [23]. Polysomnography (PSG) followed standardized techniques: digital electroencephalography (EEG), electromyography, and electrooculography signals acquired with Alice 4 system. PSG electrode montage was utilized, composed of EEG sites F3 and C3 (referenced to A2) and F4 and C4 (referenced to A1). PSG data were scored manually on a small monitor, using 30-second epochs for staging and arousal detection, as well as 2- or 5-minute respiratory data. We drew parameters from sleep polysomnography, including time in bed (TIB), total sleep time (TST), sleep latency, awakening time, sleep efficiency (TST/TIB).

Sleep architecture was assessed for each 30-second epoch coded as Wake, Stage 1, Stage 2, Stage 3+4 (slow wave sleep, SWS), and Rapid Eye-Movement (REM) sleep according to criteria made by Rechtschaffen and Kales [24]. Arousals were identified according to criteria of the American Sleep Disorders Association (ASDA) 1992 [25]. We identified apnea and hypopnea as flat air flow lower than 20% and 70% of the baseline, respectively, whose amplitude was measured during the nearest preceding period of regular breathing with stable oxygen saturation. We identified Apnea-hypopnea index as total apnea and hypopnea divided by total sleep time.

## 3. Data analysis

We divided participants into two groups according to severity of clinical manifestation Cut-off value was median number of the PANSS total scores. Student’-T test compared all variables between the two groups.

## 4. Results

Age and gender between groups were similar, as was duration of education and age at illness onset. Duration of illness of the H-PANSS group was longer. Clinical Global Impression (CGI) [11] tallied higher and Quality of Life Scale (QLS) lower in the H-PANSS group, depressive symptoms rated by Hamilton Depression Scale similar between groups (Table 1). Current medications calculated by chlorpromazine and Diazepam equivalents were also similar. There were no differences between the two groups in severity of EPS rated by Abnormal Involuntary Movement Scale, Barnes Akathsia Rating Scale, and Simpson-Angus Scale (Table 1).

**Table 1.** Demographic and clinical characteristics

Demographic characteristics	L-PANSS (n=80)	H-PANSS (n=9)	P value
Age (years)	35 ± 9.3	37 ± 9.6	0.801
Male/Female(male percentage)	2/6 (25%)	4/5 (44%)	0.434
Duration of education (years)	13.0 ± 3.3	11.6 ± 3.6	0.405
Age at illness onset (years) (years)	25.1 ± 7.5	22.3 ± 7.4	0.453
Duration of illness (months)	101.5 ± 71.8	186.7 ± 63.8	0.021 ^*^
**Clinical psychiatric condition rating scales**			
Clinical Global Impression (CGI)	2.9 ± 0.4	3.7 ± 0.5	0.002 ^**^
Quality of life scale (QLS)	63.3 ± 11.4	35.4 ± 14.0	< 0.001^**^
Hamilton Depression Rating scale	8.1 ± 5.2	12.7 ± 8.4	0.207
**Medication amount**			
Chlorpromazine equivalents	212.5 ± 64.1	237.2 ± 91.0	0.532
Diazepam equivalents	12.5 ± 16.9	5.0 ± 6.1	0.232
**Extra-pyramidal symptoms rating scales**			
Abnormal Involuntary Movement scale	4.1 ± 6.3	5.11 ± 4.5	0.712
Barnes Akathisia Rating scale	0.6 ± 1.2	2.1 ± 2.6	0.162
Simpson-Angus scale	5.8 ± 4.7	7.3 ± 3.5	0.440

**All data were expressed as mean value ± standard deviation, except gender**.

**Low-PANSS (L-PANSS) group included schizophrenics with Positive and Negative Syndrome Scale (PANSS) total score below 65 (median of PANSS total scores of all 17 subjects); High-PANSS (H-PANSS) group included those with PANSS total scores 65 or higher**.

^*^:**P<0.05 and** ^**^:**P<0.01, significance between groups**.

Cognitive performances between groups were similar. (Table 2)

**Table 2.** Cognition tests measured in two groups of patients

Parameters	L-PANSS (n=8)	H-PANSS (n=9)	P value
Trail making test	1.6 ± 0.9	2.0 ± 1.0	0.435
Digit symbol coding	4.9 ± 2.0	4.6 ± 3.1	0.807
Semantic association of verbal fluency	0.6 ± 0.5	0.4 ± 0.5	0.488
Maze	3.8 ± 1.6	4.2 ± 4.6	0.787
Verbal working memory	7.6 ± 3.9	8.7 ± 3.9	0.590
Non-verbal working memory	4.5 ± 3.1	5.6 ± 3.1	0.491
Instant word list	5.6 ± 2.8	5.1 ± 3.0	0.719
Delay word list	6.5 ± 2.6	6.6 ± 2.2	0.963
Instant visual reproduction	4.9 ± 2.2	4.8 ± 2.3	0.931
Delay visual reproduction	6.4 ± 2.6	5.1 ± 2.2	0.291

**Data were expressed as mean value ± standard deviation**.

**Low-PANSS (L-PANSS) group included schizophrenics with Positive and Negative Syndrome Scale (PANSS) total score below 65 (median of PANSS total scores of all 17 subjects); High-PANSS (H-PANSS) group comprised those with PANSS total scores 65 or higher. No significance appeared between groups**

Body weight and neck circumference (NC) in the H-PANSS group were higher than those in the L-PANSS group. Body height, BMI, waist circumference, hip circumference, WHR and body fat between groups were similar, as was physical fitness measured by sit-up and climbing (Table 3). Both systolic and diastolic blood pressures in the H-PANSS group were higher. Metabolic index, heart rate, fasting sugar, insulin, Homa-IR, cortisol, cholesterol, triglyceride, high-density lipoprotein, and low-density lipoprotein between groups were similar (Table 3).

**Table 3.** Cardiometabolic parameters and physical fitness

Physical parameters	L-PANSS (n=8)	H-PANSS (n=9)	P value
Body weight (BW) (kg)	65.4 ± 9.6	78.3 ± 9.2	0.013^*^
Body height (BH) (cm)	144.9 ± 12.5	161.1 ± 15.3	0.031^*^
Body mass index (BMI) (kg/m2)	31.5 ± 5.5	30.5 ± 4.1	0.653
Neck circumference (NC) (cm)	35.0 ± 2.6	38.6 ± 2.1	0.007^**^
Waist circumference (cm)	91.5 ± 10.5	95.8 ± 9.2	0.386
Hip circumference (cm)	104.4 ± 9.1	106.8 ± 6.5	0.537
Waist-hip ratio (WHR)	0.89 ± 0.07	0.87 ± 0.05	0.652
Body fat (%)	33.8 ± 5.1	32.1 ± 6.7	0.564
**Physical fitness**			
Sit-up (/min)	14.5 ± 12.7	14.2 ± 9.4	0.959
Stair-climbing PEHR 1 (/min)	52.9 ± 10.4	53.1 ± 8.7	0.960
PEHR 2 (/min)	48.4 ± 9.6	47.9 ± 8.5	0.913
PEHR 3 (/min)	47.0 ± 8.2	45.8 ± 8.3	0.765
Climbing time (s)	114.8 ± 57.4	133.3 ± 39.2	0.443
**Cardiometabolic parameters**			
Heart rate (/minute)	83.3 ± 16.8	85.9 ± 6.9	0.671
Systolic blood pressure (mmHg)	107.8 ± 8.2	122.7 ± 6.3	< 0.001^**^
Diastolic blood pressure (mmHg)	65.8 ± 7.1	77.8 ± 10.2	0.014^*^
Fasting sugar (mg/dL)	91.1 ± 9.6	100.7 ± 17.7	0.196
Insulin (uIU/mL)	9.62 ± 4.04	29.30 ± 54.06	0.322
Homa-IR	2.21 ± 1.01	8.48 ± 17.04	0.316
Cortisol (ug/dL)	13.4 ± 2.4	11.4 ± 4.9	0.309
Total cholesterol (mg/dL)	202.6 ± 35.0	215.1 ± 44.3	0.532
Triglyceride (mg/dL)	228.5 ± 164.3	144.6 ± 77.5	0.190
High-density lipoprotein (mg/dL)	43.9 ± 16.0	41.2 ± 7.2	0.661
Low-density lipoprotein (mg/dL)	115.0 ± 27.9	142.4 ± 37.4	0.111
Metabolic syndrome index	1.4 ± 1.1	1.8 ± 1.6	0.549^:^

**All data were expressed as mean value ± standard deviation**.

**Low-PANSS (L-PANSS) group included schizophrenics with Positive and Negative Syndrome Scale (PANSS) total score below 65 (median of PANSS total scores of all 17 subjects); High-PANSS (H-PANSS) group included those with PANSS total score 65 or higher. PEHR1, 2, 3: post-exercise heart rate in the first, second and third minute, Homa-IR: homeostasis model assessment of insulin resistance**

^*^:**P<0.05 and** ^**^:**P<0.01, significance between groups**.

The mean scores of respective sleep questionnaires, including ESS, ISI, PAS, and PSQI, were similar between L-PANSS and H-PANSS groups (Table 4). Parameters of sleep continuity measured by PSG, including awakening time, bed time, sleep efficiency, sleep latency, and total sleep time between groups were all similar. Marginal difference between the two groups were noted in the ratio of stage 3 and 4 sleep (slow wave sleep) and oxygen saturation rates.

**Table 4.** Sleep parameter measurement

Sleep continuity	L-PANSS (n=8)	H-PANSS (n=9)	P value
Awakening time	7.0 ± 0.9	7.2 ± 0.4	0.673
Bed time	22.7 ± 1.8	20.9 ± 1.6	0.052
Sleep efficiency (%)	84.0 ± 12.0	63.0 ± 28.3	0.071
Sleep latency	30.9 ± 20.2	24.3 ± 19.4	0.505
Total sleep time	7.5 ± 0.7	8.4 ± 1.1	0.067
**Sleep questionnaires**			
Epworth Sleepness Scale	9.0 ± 5.2	8.0 ± 4.0	0.663
Insomnia Severity Index	9.1 ± 3.7	8.3 ± 3.2	0.646
Pre-Sleep Arousal Scale	32.9 ± 16.6	26.4 ± 11.5	0.363
Pittsburgh Sleep Quality Index	13.8 ± 7.1	14.3 ± 7.5	0.872
**Sleep architecture**			
NREM S1 (%)	12.5 ± 14.6	29.3 ± 3.5	0.190
NREM S2 (%)	62.8 ± 19.0	47.0 ± 21.3	0.129
NREM S3 + 4 (%)	8.0 ± 9.1	1.1 ± 2.9	0.047^*^
REM sleep (%)	16.6 ± 5.5	22.7 ± 16.6	0.337
**Sleep obstruction parameters**			
Apnea-hypopnea index	6.2 ± 8.8	8.8 ± 9.4	0.560
Mean SpO2 (%)	96.6 ± 1.5	95.0 ± 1.5	0.046^*^
ALM (events/hour)	8.8 ± 8.1	19.4 ± 19.0	0.164
Leg movement	53.1 ± 109.2	11.6 ± 34.7	0.295

**All data were expressed as mean value ± standard deviation**.

**Low-PANSS (L-PANSS) group included schizophrenics with Positive and Negative Syndrome Scale (PANSS) total score below 65 (median of PANSS total scores of all 17 subjects); High-PANSS (H-PANSS) group included those with PANSS total scores 65 or higher. NREM: non-rapid eye movement, REM: rapid eye movement, SpO2: saturation of peripheral oxygen, ALM: arousal and limb movement. NREM S3 + 4 (SWS) in the H-PANSS group was lower. Intergroup NREM S1, S2 and REM sleep were similar (Table** **4**). **Mean SpO2 in the H-PANSS group was lower. Apnea-hypopnea index, Arousal and Limb Movement, and leg movement between groups were similar (Table** **4**).

^*^:**P<0.05 and** ^**^:**P<0.01, significance between groups**.

## 4. Discussion

To our knowledge, this is the first study to suggest that severer clinical symptoms are associated with metabolic and sleep disturbance in patients with schizophrenia. In more detail, this study demonstrates that schizophrenia patients with severe symptomatology may have more metabolic abnormalities including heavier body weight, wider neck circumference, and elevated systolic/diastolic blood pressure. We found no intergroup statistical significance in terms of blood sugar, insulin, cortisol, and lipid profiles. This is the first study to suggest that schizophrenic patients with more severe symptoms might have decreased oxygen saturation. It also demonstrated that patients with more severe symptoms had reduced SWS when their sleep efficiency and total sleep time were similar to the low PANSS group. Results concurred with prior studies: positive symptoms of schizophrenia increased REM sleep eye movement density, short REM latency, reduced sleep efficiency and prolonged sleep latency [26], [27], [28], [29]. Conversely, negative symptoms relate to short REM latency and SWS deficit [30], [31]. cognitive symptoms to SWS deficit [28], [29]. Sarkar et al. [32] found significant difference in SWS parameters (including increased Stage 3 and decreased Stage 4 latency between patients and controls.

The strength of this study is control over two groups of patients similar in basic demographic data, cognitive function performance, and physical fitness. Limitations of the study included small sample size and cross-section design. In sum, this study suggests clinical symptoms as linked with heavier body weight, wider neck circumference, elevated blood pressure, and shorter SWS in schizophrenic pateints. Further studies must confirm preliminary findings and elucidate the underlying mechanism.

## Acknowledgments

This study is supported in part by Taiwan Department of Health Clinical Trial and Research Center of Excellence (DOH103-TD-B-111-004).
